# Deciphering the physiopathology of neurodevelopmental disorders using brain organoids

**DOI:** 10.1093/brain/awae281

**Published:** 2024-09-02

**Authors:** Olivier Dionne, Salomé Sabatié, Benoit Laurent

**Affiliations:** Research Center on Aging, Centre Intégré Universitaire de Santé et Services Sociaux de l'Estrie-Centre Hospitalier Universitaire de Sherbrooke, Sherbrooke, QC J1H 4C4, Canada; Research Center on Aging, Centre Intégré Universitaire de Santé et Services Sociaux de l'Estrie-Centre Hospitalier Universitaire de Sherbrooke, Sherbrooke, QC J1H 4C4, Canada; Research Center on Aging, Centre Intégré Universitaire de Santé et Services Sociaux de l'Estrie-Centre Hospitalier Universitaire de Sherbrooke, Sherbrooke, QC J1H 4C4, Canada; Department of Biochemistry and Functional Genomics, Faculty of Medicine and Health Sciences, Université de Sherbrooke, Sherbrooke, QC J1H 5H4, Canada

**Keywords:** organoids, induced pluripotent stem cells, 3D culture, brain, neurodevelopmental disorders

## Abstract

Neurodevelopmental disorders (NDD) encompass a range of conditions marked by abnormal brain development in conjunction with impaired cognitive, emotional and behavioural functions. Transgenic animal models, mainly rodents, traditionally served as key tools for deciphering the molecular mechanisms driving NDD physiopathology and significantly contributed to the development of pharmacological interventions aimed at treating these disorders. However, the efficacy of these treatments in humans has proven to be limited, due in part to the intrinsic constraint of animal models to recapitulate the complex development and structure of the human brain but also to the phenotypic heterogeneity found between affected individuals. Significant advancements in the field of induced pluripotent stem cells (iPSCs) offer a promising avenue for overcoming these challenges. Indeed, the development of advanced differentiation protocols for generating iPSC-derived brain organoids gives an unprecedented opportunity to explore human neurodevelopment.

This review provides an overview of how 3D brain organoids have been used to investigate various NDD (i.e. Fragile X syndrome, Rett syndrome, Angelman syndrome, microlissencephaly, Prader-Willi syndrome, Timothy syndrome, tuberous sclerosis syndrome) and elucidate their pathophysiology. We also discuss the benefits and limitations of employing such innovative 3D models compared to animal models and 2D cell culture systems in the realm of personalized medicine.

## Teaser

Dionne *et al.* review the use of 3D brain organoids to study neurodevelopmental disorders and their underlying pathophysiology. They discuss the benefits and limitations of using these 3D models compared to animal models and 2D cell culture systems, particularly in the realm of personalized medicine.

## Introduction

### Development of the human cortex

**Figure 1:**
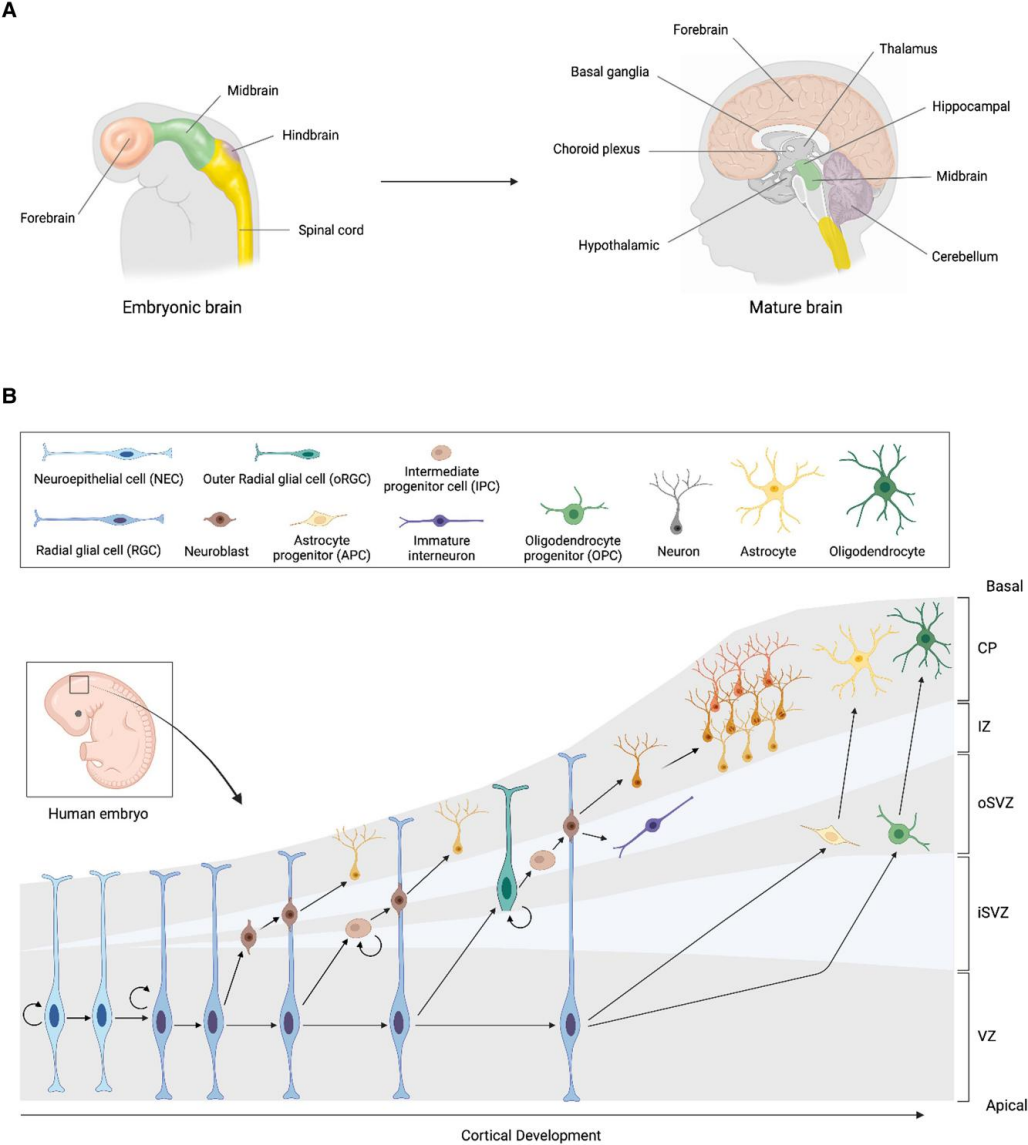
**Human brain development**. (**A**) Schematic representation of some embryonic brain regions (forebrain, midbrain, hindbrain, spinal cord) and their corresponding regions in human adult brain with matching colours. (**B**) Schematic of the key cellular processes involved in human cortical development, which include expansion of the neuroepithelial cell (NEC) pool, radial glial cell (RGC) differentiation, neurogenesis, neuronal precursor radial and tangential migration, maturation of immature neurons, cortical layers formation and gliogenesis. CP = cortical plate; IZ = intermediate zone; oSVZ = outer subventricular zone; iSVZ = inner sub-ventricular zone; VZ = ventricular zone.

Brain development requires the precise coordination of multiple tightly regulated events. It starts with neurulation; the process during which the neural plate of the embryonic ectoderm folds and fuses to form the neural tube.^1^ The neural tube is subsequently segmented through various patterning processes, enabling the development of all regions within the CNS (Fig. 1A).^2^ The ventricular zone (VZ), situated at the apical surface of the neural tube, is inhabited by neuroepithelial cells (NECs), which stand as the multipotent stem cells of the nervous system.^3^ NECs initially undergo a period of proliferation to allow the expansion of the stem cell pool.^1,4^ When corticogenesis begins, NECs differentiate into radial glial cells (RGCs), which are neural progenitors responsible for generating neuronal and glial cells (Fig. 1B).^1,4^ RGCs exhibit a distinct polarized morphology characterized by an apically localized soma and long radial processes that extent seamlessly from the apical membrane to the pial surface.^5,6^ Like NECs, RGCs initially undergo a period of symmetric, self-renewing divisions to amplify the pool of progenitor cells.^7,8^ At the onset of cortical neurogenesis, RGCs also begin to undergo asymmetric division, leading to the generation of a neuron and another RGC in a process known as direct neurogenesis.^9^ The asymmetrical division of RGCs can also result in the formation of intermediate progenitor cells (IPCs), which populate the subventricular zone (SVZ) and are characterized by their limited proliferative capacity (Fig. 1B). Two neurons will ultimately be generated from a single IPC. This process, known as indirect neurogenesis, enables the amplification of neuronal output from the progenitor cell pool.^9-11^ Later in cortical development, RGCs also generate a second type of progenitor known as outer radial glial cells (oRGCs), which reside in the outer region of the SVZ (Fig. 1B).^12^ Notably, unlike RGCs, oRGCs lack attachment to the apical membrane of the VZ, but maintain their radial processes, which extend to the pial surface.^12,13^ Owing to their increased proliferative capacity, oRGCs become the predominant type of progenitors within the human brain around mid-gestation, taking on the responsibility of producing the majority of neurons within the human cortex.^14-17^ Neuronal differentiation and maturation occur along with migration, which allows for the integration of environmental cues during network establishment.^18,19^ Dendritic spine morphogenesis and synaptogenesis follow the formation of neuronal networks and enable communication between neurons.^20-22^ The refinement of synaptic connections, which mainly occurs through dendritic and synaptic pruning, persists into adulthood.^20^ Around mid-gestation, RGCs and oRGCs cease the generation of neurons and transition to gliogenesis. This process leads to the formation of astrocyte progenitor cells (APCs) and oligodendrocyte progenitor cells (OPCs) (Fig. 1B). These progenitors later undergo differentiation, giving rise to mature astrocytes and oligodendrocytes, which then become integrated into neuronal circuits.^20,23-25^ These glial cells play a crucial role in supporting the functions of neurons.^26-29^

### Neurodevelopmental disorders

Neurodevelopmental disorders (NDD) refer to a group of conditions characterized by abnormal brain development and impaired cognitive, emotional and behavioural functions.^30^ Approximately 4.7% of children worldwide are affected by NDD.^31^ The clinical manifestations of NDD vary greatly across disorders and even among individuals with the same condition. Common features include intellectual disability, autism spectrum disorder (ASD) and various developmental delays.^30,31^ The causes of NDD are complex and multifactorial, involving a combination of genetic and environmental factors.^32,33^ The use of animal models has been instrumental in understanding the underlying molecular mechanisms that contribute to the cognitive and behavioural impairments observed in NDD. Transgenic rodent models have facilitated the advancement of potential pharmacological interventions for treating these disorders. However, the translational value of these findings has been hindered significantly by the limitations associated with these animal models.^34,35^ One key restraint comes from disparities between rodents and humans in terms of brain structures, functions and development, limiting the extent to which animal models can accurately replicate the human pathophysiology of NDD.^4^ Moreover, transgenic animals lack the ability to replicate the intrinsic genetic diversity present in human populations, which is crucial for capturing the complete range of phenotypes observed in NDD.^33,36^ Finally, many pharmacological treatments shown to be promising in animal models failed to be efficient in human subjects.^37^ Collectively, these limitations underscore the necessity of employing models that reproduce NDD with higher fidelity.

### Pluripotent stem cells for modelling human brain development

**Figure 2:**
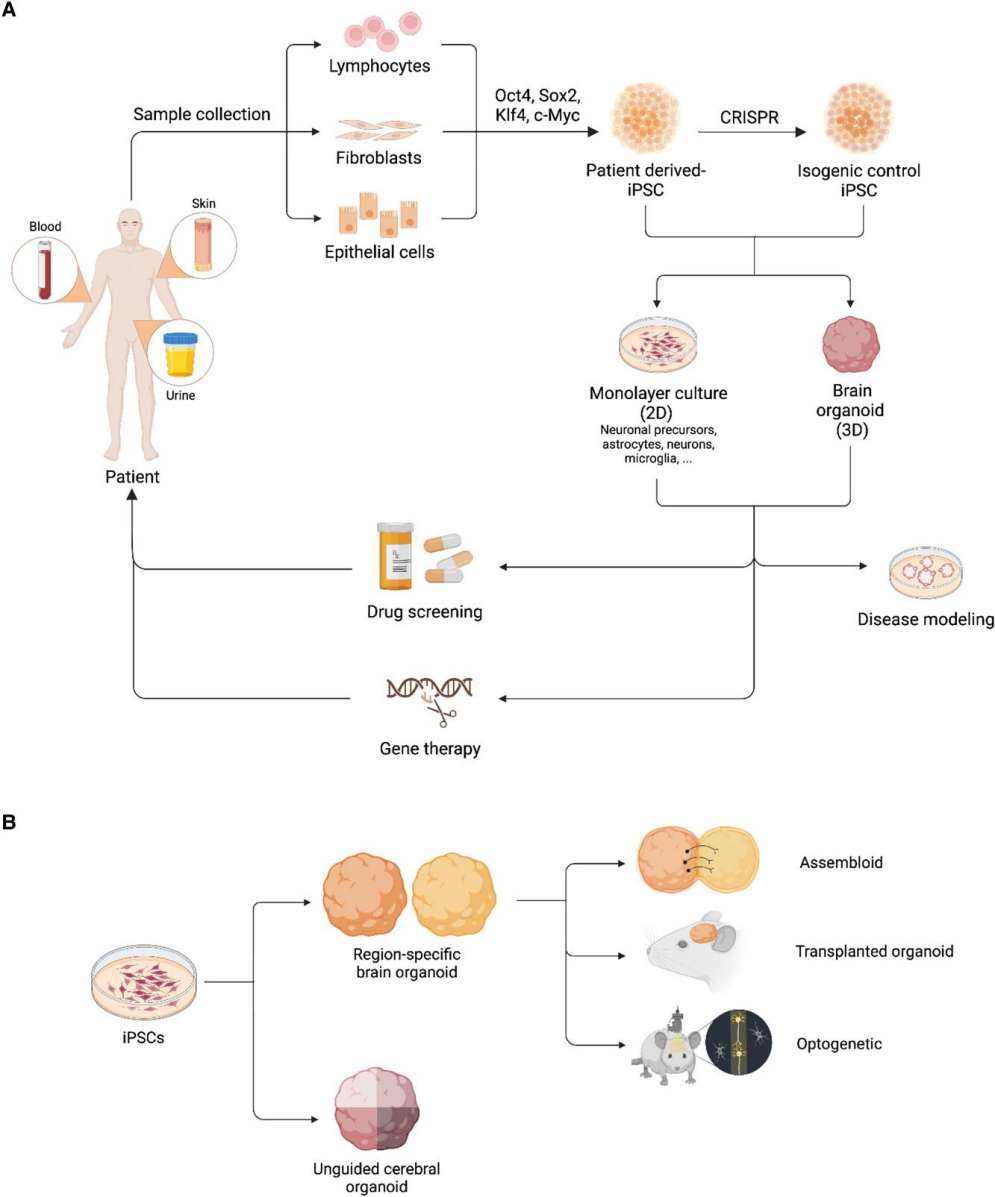
**Stem cells and organoids**. (**A**) Schematic of the different induced pluripotent stem cell (iPSC)-based systems in place for the study of neurodevelopmental disorders (NDD). Various somatic cell types can undergo reprogramming to generate iPSCs. Using both 2D and 3D iPSC-based models allows for the examination of NDD physiopathology, as well as conducting drug screening assays. These patient-derived iPSCs can be edited and further used for potential regenerative cell therapy applications. (**B**) Schematic of how iPSCs can be used to generate unguided or region-specific human brain organoids, including innovative approaches such as the creation of assembloids, the transplantation of human brain organoids in animal models and the application of optogenetics. CRISPR = clustered regularly interspaced short palindromic repeats.

Human pluripotent stem cells (hPSCs) are a valuable resource for the *in vitro* investigation of human brain development and its related diseases. There are two categories of hPSCs: induced pluripotent stem cells (iPSCs) and embryonic stem cells (ESCs). ESCs are derived from the inner mass cells of the blastocyst,^38,39^ whereas iPSCs are generated by reprogramming adult somatic cells isolated and cultured from skin, urine or blood samples. These somatic cells are reprogrammed into iPSCs using specific transcription factors (i.e. Oct4, Klf4, Sox2, c-Myc) (Fig. 2A). These cells possess remarkable abilities to self-renew and potentially differentiate into various specialized cell types, making them a powerful tool for studying NDD physiopathology, for screening drugs and developing gene therapy strategies (Fig. 2A).^40^ Various protocols have been established to differentiate hPSCs into different types of brain cells within a monolayer culture system, and extensively utilized in NDD research. However, these two-dimensional models harbour intrinsic limitations such as a lack of spatial complexity, limited diversity of cell types and an inability to replicate crucial processes, e.g. cell migration, cell polarization and complex cell–cell interactions.^41^

Brain organoids are complex cellular structures that closely mimic the development of the human brain. They exhibit similar cytoarchitecture, gene expression patterns and epigenomic characteristics.^42,43^ These organoids are created by allowing hPSCs to aggregate and form embryoid bodies. Embryoid bodies are then cultivated in a specific medium to guide them towards ectodermal tissue differentiation. When cultured under appropriate conditions, they develop into rosettes that resemble the neural tube and contain neural progenitor cells (NPCs). These NPCs continue to proliferate and expand the organoid epithelia before undergoing neurogenesis and gliogenesis phases. The cell diversity and structure of these organoids depend on the morphogens used during their initial formation. Indeed, unguided protocols result in organoids (called cerebral organoids) composed of various regionally defined structures, whereas guided protocols use specific morphogens to specify the regional identity of the progenitor cells (Fig. 2B).^44,45^ Through the years, various brain organoids have been developed to further study different adult brain regions such as choroid plexus, hindbrain or forebrain (Fig. 1A).^45,46^ Moreover, interaction between brain regions can be modelled by fusion organoids (also known as assembloids), which are created by fusing two organoids with distinct regional identities (Fig. 2B). Brain organoids can also be transplanted into rodent brains to promote vascularization and neuronal circuit maturation (Fig. 2B).^4^

Brain organoids have emerged as a powerful tool that addresses the limitations of animal models and monolayer culture systems to study human brain development. This review aims to present a summary of recent studies that have employed organoids to investigate different NDD.

## Fragile X syndrome

Fragile X syndrome (FXS) is the leading cause of intellectual disability, affecting one in every 2500–4000 males and one in every 7000–8000 females.^47^ Affected individuals are also at risk of developing a wide array of psychiatric and behavioural comorbidities such as ASD, anxiety, hyperactivity, hyperarousal and attention deficit.^48^ FXS is caused by the absence of expression of the Fragile X messenger ribonucleoprotein (FMRP), resulting from the silencing of the *FMR1* gene. *FMR1* transcription is absent in FXS individuals due to the hypermethylation of its promoter region. This epigenetic modification happens when there is an expansion of more than 200 CGG repeats at the 5′-untranslated region in the offspring of mothers who carry a premutation (55–200 CGG repeats) without any abnormal methylation.^49,50^ FMRP is an RNA-binding protein whose main function is to regulate the translation of its mRNA targets in many tissues, including the brain.^51-53^ As such, loss of FRMP translational control has been linked repeatedly to abnormal protein synthesis.^54-58^ Previous research has shown that this defect drives the neuronal hyperexcitability observed in FXS animal models through exaggerated metabotropic glutamate receptor 5 (mGluR5) signalling and reduced GABAergic inhibition.^59,60^ Hence, inhibition of mGluR5 or enhancement of the GABAergic system have been shown to correct core behavioural and cellular deficits in FMR1 knockout (KO) animals.^61,62^ However, clinical trials using mGluR5 inhibitors and agonists of the GABAergic system have been deemed unsuccessful.^63,64^ The failure to efficiently translate findings in animal models to FXS patients highlights the need to investigate the pathophysiology using human models.

Several research groups have undertaken this endeavour using brain organoids. Brighi *et al*.^65^ used cerebral organoids derived from FMR1-KO iPSCs to identify anatomical defects induced by FMRP deficiency. Indeed, they showed that FMR1-KO cerebral organoids were larger than their isogenic counterpart (at both mid and late time points during organoid differentiation) and harboured more structures resembling cortical plates. They also showed that FMRP-deficient organoids display a higher number of GFAP-positive cells, which are likely astrocytes. Interestingly, a recent study also reported a higher number of astrocytes in post-mortem cortices of FXS individuals.^66^ Molecular defects were also revealed using FXS 3D culture. For example, Cencelli *et al*.^67^ measured higher expression of soluble amyloid precursor protein alpha (sAPPα) in forebrain organoids derived from FXS iPSCs. Overexpression of sAPPα is linked to alterations typically associated with the FXS phenotype, such as aberrant spine morphogenesis, dysregulated protein synthesis and abnormal synaptic transmission.^68,69^ Based on this observation, further studies should explore the potential contribution of sAPPα dysregulation to FXS physiopathology.

Brain organoids have also been utilized to investigate neurodevelopmental defects induced by FMRP absence. In FXS forebrain organoids, NPC proliferation is increased at Day 28 and decreased at Day 56.^70,71^ The ability of progenitors to differentiate is also hindered by FMR1 silencing. Indeed, FXS forebrain organoids exhibit altered cytoarchitecture with a thinner VZ and a thicker cortical plate. Furthermore, neuronal differentiation is accelerated and associated with abnormal neuronal specification, characterized by an increased number of excitatory neurons and a reduced number of inhibitory neurons. Moreover, single-cell RNA-sequencing (scRNA-seq) analysis revealed that FXS forebrain organoids present an expanded population of RGCs and a smaller population of inhibitory neurons with a more immature signature. Altogether, these defects were shown to increase synapse formation and enhance neuronal excitability in FXS organoids.^71^

Human-specific features of FXS have also been uncovered using 3D brain models. By conducting enhanced crosslinking and immunoprecipitation (eCLIP) on forebrain organoids, Kang *et al*.^71^ have identified over 3500 high-confidence RNA targets of FMRP. Notably, around 66% of these transcripts were exclusively human-specific, distinguishing them from the eCLIP results obtained from mouse fetal brain. Interestingly human-specific RNA targets of FMRP were shown to be associated with the Wnt pathway and synaptic signalling, whereas mouse-specific targets are linked to glutamate receptor signalling.^71^ In the same study, the researchers demonstrated that treating FXS forebrain organoids with phosphoinositide 3-kinase (PI3K) inhibitors, but not mGluR5 inhibitors, effectively normalized progenitor cell proliferation and synapse formation.^71^ These findings indicate that brain organoids can serve as a model for identifying human-specific pathophysiological features and assessing the therapeutic potential of pharmacology in FXS.

## Rett syndrome

Rett syndrome (RTT) is one of the most common causes of intellectual disability and predominantly results from loss-of-function mutations within the *MECP2* gene, located on the X chromosome, which encodes methyl-CpG binding protein 2 (MeCP2).^72,73^ RTT is estimated to affect one in every 10 000 to 15 000 females and is hardly ever diagnosed in males.^74,75^ In humans, RTT is characterized by developmental stagnation and regression, starting between 6 and 18 months of age. During this period, patients gradually lose the ability to perform several skills such as walking, language, and social interaction and experience degradation of their general motor coordination, which ultimately impedes their quality of life and poses a significant burden for their caregivers.^76,77^ MeCP2 is strongly expressed in brain where it binds methylated cytosine in DNA to negatively modulate transcription.^78,79^ Studies in animal models showed that disruption of MeCP2-mediated transcription affects the expression of a wide array of messenger and non-coding RNAs, which are tough to lead to the abnormal neurogenesis and neural circuit formation observed in RTT.^80-83^ However, the precise molecular mechanisms underlying these deficits remain to be fully elucidated.

Brain organoids have been employed on multiple occasions to study the neurodevelopmental abnormalities caused by the loss of MeCP2 function. In fact, a decrease in the number of NPCs in RTT cerebral and cortical organoids has been observed and correlated with a reduction in SVZ thickness.^84-86^ Additionally, MeCP2 was found to play a role in regulating neural cell proliferation, as shown by the decreased number of dividing cells and the abnormal cell cycle progression observed in RTT organoids.^84,85,87^ Intriguingly, MePC2 deficiency also affects the orientation of the mitotic spindle.^85^ This latter finding suggests that MECP2 mutation may dampen NPC differentiation. Consequently, neurogenesis was consistently shown to be diminished in RTT organoids, with a related increase in gliogenesis. Furthermore, the composition of neuronal subpopulations was influenced by MeCP2, resulting in an elevated number of glutamatergic neurons, a reduced number of GABAergic neurons in RTT organoids and a concomitant increased neuronal hyperexcitability.^85-87^

Fusion organoids serve as models to enable the exploration of interactions among different embryonic brain regions. In this sophisticated system, organoids of specific brain regions are initially produced independently and then assemble after a period of maturation (Fig. 2B).^88^ For instance, the fusion of ganglionic eminence (GE) and cortex (Cx) organoids (referred to here as GE-Cx) enables the examination of interneuron migration from the GE, the integration of inhibitory neurons and the formation of inhibitory synapses in Cx organoids.^89^ The evaluation of GE-Cx organoid electrophysiological activity has uncovered the presence of simultaneous sustained oscillations at multiple frequencies, a feature of mature neural networks difficult to observe in traditional brain organoid models.^90,91^ Fusion organoids were used to gain a deeper understanding of the alterations previously identified in RTT cerebral and cortical organoids. Indeed, scRNA-seq analysis of fusion organoids has unveiled changes in the composition of inhibitory neuron populations induced by MECP2 loss-of-function. Differential gene expression analysis has also highlighted dysregulation of transcripts associated with neuronal projection and synaptic formation.^91^ Consequently, RTT GE-Cx organoids exhibit abnormal electrophysiological activity, characterized by neuronal hyperexcitability and atypical neural oscillation patterns. Intriguingly, these anomalies were absent in fusion organoids constructed from control GE organoids and RTT Cx organoids, suggesting that the dysfunctions are driven primarily by interneuron abnormalities.^91^ Indeed, reduced interneuron migration has been demonstrated in RTT fusion organoids.^87^

There is currently no known remedy for RTT. Nevertheless, the US Food and Drug Administration recently granted approval to use trofinetide as a treatment for RTT. Nevertheless, there is still limited understanding regarding trofinetide’s mode of action and whether its administration would be beneficial for all RTT individuals.^92^ The identification of new pharmacological interventions, therefore, remains a priority research area for the field. Brain organoids have been used as a platform to screen for potential drug candidates in the context of RTT. Indeed, Trujillo *et al*.^93^ used a combination of 2D and 3D iPSC-based models to investigate the ability of 14 molecules to correct abnormal synaptic transmission resulting from MeCP2 deficiency.^93^ During a first screening phase using 2D-cultured neurons, they selected two of the 14 initials drug candidates (nefiracetam and PHA 543613) based on their ability to restore the number of synaptic puncta and electrophysiological activity measured by multielectrode array (MEA) recording. Using cortical organoids, they further confirmed the efficacy of these pharmacological agents by showing that organoid treatment with both molecules increased the expression of genes related to synaptogenesis and neurotransmitter metabolism to normalize neural network activity.^93^ This study constitutes a proof-of-concept that drug-screening platforms based on both 2D and 3D iPSC-derived models can be relevant tools for translational research in the context of NDD.

Other studies have also investigated the ability of other drug candidates to directly target precise molecular deficits exhibited by RTT brain organoids. For example, activation of the PI3K/AKT pathway in cerebral organoids by a valproic acid treatment rescued the reduced number of proliferating cells, increased neuron generation and partially normalized gene expression.^84^ Investigations on RTT cortical organoids revealed upregulation of the TGF-β/BMP signalling pathway, which plays a pivotal role in ectodermal fate determination and subsequent NPC differentiation. Inhibition of this pathway using LDN193189 or dorsomorphin was able to restore neuron specification by reducing the production of glutamatergic neurons and increasing the number of GABAergic neurons while concomitantly decreasing gliogenesis.^85^ Inhibition of p53 using pifithrin-α was also shown to be effective in rescuing the abnormal neural network oscillation in GE-Cx fusion organoids.^91^

## Microlissencephaly

The human cerebral cortex is made up of a complex arrangement of creases (sulci) and ridges (gyri) known as gyrification. This intricate folding pattern increases the cortex surface area, which allows for the establishment of a greater number of neural connections. Gyrification plays a crucial role in enhancing cognitive functions through improved connectivity and specialization of various cortical regions.^94,95^ The process of human brain gyrification initiates around the 16th week of gestation and continues until the 40th week.^96,97^ However, many critical cellular events essential for this developmental process, such as the expansion of progenitor cells and neuronal migration, begin as early as the fourth week of gestation.^98,99^ Disruptions in these cellular mechanisms can hinder the formation of gyrification in the brain, leading to impaired cognitive functions in affected individuals. Microlissencephaly represents a spectrum of cortical development disorders characterized by abnormal cortical folding, reduced brain size and severe clinical symptoms, including feeding difficulties, intellectual disability and delayed motor development.^100^ Over recent decades, mutations in various genes have been identified as causal factors for lissencephaly.^101^ Understanding the mechanisms connecting these genetic dysfunctions to abnormal gyrification has been challenging when using animal models, primarily because the brains of mice and rats are naturally lissencephalic.^99^ Organoid-based models hold significant relevance for investigating this neurodevelopmental condition. They accurately replicate the timing and cellular processes involved in gyrification. However, it is important to emphasize that brain organoids can only generate gyrification under specific physical conditions and do not naturally exhibit this feature using typical protocols.^102,103^

Miller-Dieker syndrome (MDS) is the most severe form of microlissencephaly and is associated with several cognitive impairments.^104^ MDS arises from deletions within the 17p13.3 locus, which encodes the *LIS1* and *YWHAE* genes, two members of a multi-protein complex crucial for the regulation of microtubule dynamics, centrosomal protein localization and proliferation of RGCs.^99,105,106^ The generation of cerebral organoids using iPSCs derived from MDS patients has allowed the identification of several neurodevelopmental abnormalities. The SVZ of MDS organoids exhibits a disrupted architecture, characterized by loosely arranged cells randomly positioned in relation to the apical membrane and disorganized adherence molecules.^107^ Additionally, MDS organoids are smaller in size, mirroring the microencephaly seen in patients.^107,108^ A comprehensive analysis of MDS organoids revealed an increased expression of the apoptotic marker caspase 3 but no alterations in the expression of the proliferation marker Ki67.^108^ Mitotic orientation is also disrupted, favouring a horizontal cleavage plane (asymmetric division), resulting in an increased number of TBR2-positive IPCs in patient organoids.^107,108^ These findings suggested that the microencephaly observed in MDS patients may originate from a reduced pool of stem cells. Furthermore, the microtubule network of RGC, which composes the radial scaffold essential for the migration of neuronal precursors from the SVZ to the cortical plate, was found to be incompletely expanded in MDS organoids.^107-109^ Consequently, live-cell imaging demonstrated reduced neuronal migration in MDS organoids.^108^ Interestingly, re-expression of LIS1 and/or YWHAE successfully reversed most of the aforementioned abnormalities, underscoring the critical roles of these two genes in proper RGC function during brain development.^107,108^

Katanin is a microtubule-severing protein playing a crucial role in cell motility, mitosis and neuronal morphogenesis through its ability to regulate the dynamics and organization of microtubules.^110^ Katanin is a heterodimer of p60 (encoded by the *KATNA1* gene), which exhibits ATPase enzymatic activity, and p80 (encoded by the *KATNAB1* gene), responsible for centrosome targeting.^111^ Mutations within KATNB1 are associated with severe cases of microlissencephaly.^112,113^ Cerebral organoids derived from iPSCs harbouring mutations within the KATNB1 gene were used to investigate the neurodevelopmental defects induced by p80 loss-of-function.^114^ Histological analyses revealed that mutant organoids exhibited a deformed SVZ, characterized by an irregular shape and reduced lumen size. Neurogenesis was also reduced by KATNB1 mutation, as shown by the reduced neuronal population and improper neuronal migration.^114^ Taken together, these results suggested the critical role of the Katanin p80 subunit for proper cortical development and provided insight into the contribution of its dysfunctions to the pathogenesis of microlissencephaly.

## Timothy syndrome

Timothy syndrome (TS) is a rare multisystemic disorder that results from gain-of-function mutations within the *CACNA1C* gene, which encodes the alpha-1 subunit of voltage-dependent calcium channels.^115^ TS causal mutations lead to faulty inhibition of voltage-dependent channels, which results in heightened cellular excitability due to increased intracellular calcium levels.^116^ CACNA1C is highly expressed in the brain, lung, heart, gastrointestinal system and smooth muscle. Consequently, individuals with TS exhibit a wide range of medical conditions, including cardiac arrhythmia, cardiomyopathy, facial dysmorphia and syndactyly.^115,117^ The average lifespan of TS patients is 2.5 years, with the most common cause of death being lethal arrhythmia. Several of the children who manage to survive beyond the age of 2.5 years continue to encounter developmental impairments, including delays in language and motor skills. Additionally, they may develop cognitive impairments such as intellectual disability and ASD.^115^

Brain organoid models have provided an unprecedented window into human neurodevelopment. However, the limited maturation within an *in vitro* culture system dampens the ability to model the full phenotypical spectrum of neurological disorders.^118^ Revah *et al*.^119^ overcame these shortcomings by introducing a groundbreaking method: the transplantation of human cortical organoids into the brains of newborn rats (Fig. 2B). This innovative approach allowed for the enhanced maturation and integration of transplanted cortical organoids (tCO) within the host brain. Indeed, histological analyses performed 8 months post-transplantation revealed that tCO were colonized by rat microglia and had become vascularized. Furthermore, when compared to cortical organoids cultured *in vitro*, tCO were found to house neurons of a more mature morphology and display increased electrophysiological activity. The transplanted organoids also contained oligodendrocytes, a type of glial cells absent in organoids cultured *in vitro*. Through live imaging and optogenetic experiments, it was additionally demonstrated that tCO could be activated by the surrounding rat tissue and drive reward-seeking behaviours, thus confirming their functional integration.

The examination of tCO derived from TS iPSCs unveiled distinct functional and morphological deficiencies. Patch-clamp analysis revealed heightened electrophysiological activity, marked by an elevated occurrence of spontaneous excitatory post-synaptic currents.^119^ Furthermore, it was demonstrated that neurons in TS tCO displayed an immature morphology characterized by an increased count of dendrites, elevated dendritic spine density and reduced dendritic length.^119^ Dendritic branching was also observed to be diminished in TS tCO. Remarkably, this alteration was not detectable in organoids cultured *in vitro*, underscoring the critical role of the improved maturation achieved through transplantation.^119^

Fusion organoids combining cortex and ganglionic eminence (subpallium) revealed specific shortcomings in interneurons within the context of TS. Birey *et al*.^120,121^ demonstrated that the migration of interneurons from the ganglionic eminence to the cortex was hindered in the GE-Cx fusion organoids. Notably, interneuron migration in TS was characterized by an increased saltation frequency and reduced saltation length and speed. Further investigation indicated that these abnormalities were the result of impeded rear cell contractility. Interestingly, the abnormal interneuron migration was shown to be cell autonomous, as these deficits were also observed in fusion organoids composed of TS GE organoids and control Cx organoids but not in assemblies featuring control GE and TS Cx organoids.^120^

The mechanisms underlying the abnormal interneuron migration were also investigated using TS fusion organoids. Indeed, it was shown that the reduced saltation length can be rescued by culturing TS assembloids in a low-calcium medium or by inhibiting myosin light chain kinase (MLCK), which is a calcium/calmodulin-dependent enzyme. These findings strongly indicated that calcium-mediated processes are responsible for the reduced saltation length in TS interneurons. However, it is important to note that the saltation frequency remained unaffected under these experimental conditions, suggesting that a separate mechanism may be responsible for this aspect. Moreover, RNA-seq analysis of GE and Cx organoids revealed an upregulation of various components of the GABAergic system in TS. Consequently, pharmacological inhibition of GABAa receptors was subsequently shown to restore the saltation frequency in TS interneurons.^121^ In summary, the comprehensive results from fusion organoid studies in the context of TS have unveiled defective interneuron migration and pinpointed the underlying cellular mechanisms at play.

## Chromosome 15 imprinting disorders

Genetic imprinting is a hereditary form of epigenetic control over gene expression, leading to the selective activation of one allele based on their parental origin.^122^ Approximately 100 human genes are believed to be governed by this regulatory mechanism. These imprinted genes are primarily organized in clusters throughout the genome and encode various genes and non-coding RNAs that play crucial roles during human development.^123^ Consequently, disruptions in their expression and function can lead to pathological outcomes, known as imprinting disorders. One of the well-studied imprinted loci is the 15q11.2-q13 region, which is associated with conditions such as Angelman syndrome (AS), Prader-Willi syndrome (PWS) and chromosome 15 duplication syndromes.^124^

### Angelman syndrome

AS is a NDD which mainly arises from *de novo* mutations occurring in the maternal allele of the *UBE3A* gene, which encodes an E3 ubiquitin ligase.^125^ UBE3A is expressed biallelically during early neurodevelopment before being paternally silenced in mature neurons.^126^ The canonical function associated with UBE3A is to target specific proteins for proteasomal degradation through poly-ubiquitination. Additional studies have also suggested that UBE3A may play a role in transcriptional regulation.^127^ Consequently, loss-of-function mutations within the maternal allele of UBE3A lead to intellectual disability, seizures, developmental delays and other comorbidities.^128^ Despite a decade of research performed with animal models, the pathophysiological mechanisms linking dysfunction of the UBE3A maternal allele to the AS neurodevelopmental phenotype remain poorly understood. Consequently, certain research teams have tackled this problem by using brain organoids.

Sun *et al*.^129^ employed cortical organoids to investigate electrophysiological changes associated with AS. Their study demonstrated that UBE3A-KO organoids exhibited an elevated expression of Big Potassium (BK) ion channels and heightened neuronal excitability, as assessed using MEA. Live calcium imaging analysis also revealed the presence of spontaneous burst firing and a higher level of network synchronization in UBE3A-KO organoids, resembling the seizure phenotype observed in AS patients. Additionally, when treated with paxillin, a BK channel inhibitor, burst firing was converted into single action potentials and the increased synchronized activity was rescued.^129^ Sen *et al*.^130^ used cerebral organoids derived from AS patients to investigate the subcellular distribution of UBE3A during neural development. Notably, their findings revealed that UBE3A was predominantly located in the cytoplasm of neural stem cells (SOX2-positive) and neural progenitors (PAX6-positive). However, a shift towards a predominantly strong nuclear localization was observed as neurons matured, although immature neurons (TBR1-positive) exhibited increased expression compared to mature neurons (CTIP2- or SATB2-positive). Their results showed that this subcellular localization pattern was altered in AS organoids, since UBE3A was predominantly localized in the nucleus of neural stem cells and in the cytoplasm of neurons. Furthermore, their results indicated an elevated expression of UBE3A-AS long non-coding RNA (lncRNA), which plays a role in the silencing of the paternal allele. This increased UBE3A-AS lncRNA expression was observed starting from the eighth week of AS organoid maturation, coinciding with a reduction in *UBE3A* gene expression. As such, these findings suggest an abnormal imprinting pattern in AS organoids.^130^

### Prader-Willi syndrome

PWS arises from deletions within the paternal allele of the 15q11.2-q13 locus or from maternal chromosome 15 disomy. Clinical features include intellectual disability, severe infantile hypotonia, hyperphagia, developmental delay and behavioural problems. PWS is also characterized by several metabolic and endocrine impairments, such as growth hormone deficiency, hypogonadism and obesity.^131^ The arcuate nucleus (ARC) is a key structure within the hypothalamus, which is composed of different neuronal populations and plays a crucial role in regulating appetite and energy balance.^132^ Dysfunctions in the ARC have been linked to PWS.^133,134^ There is limited understanding of the mechanisms underlying PWS physiopathology within the developing ARC, primarily due to the lack of a reliable *in vitro* model.

Huang *et al*.^135^ successfully addressed this issue by developing a method for consistently generating ARC organoids from iPSCs. Their protocol involves an extended process of hypothalamic patterning, which begins at the outset of neural ectoderm specification. This patterning is achieved through the dual inhibition of SMAD signalling and the activation of the Sonic Hedgehog (SHH) pathway, while concurrently suppressing the Wnt pathway. The organoids are subsequently matured using hypothalamic astrocyte-conditioned medium supplemented with neurotrophic factors. Analysis of ARC organoids generated from PWS iPSCs unveiled numerous neurodevelopmental abnormalities. Specifically, PWS organoids displayed an enlarged size, an elevated number of proliferative cells and astrocytes and a diminished neuronal population. Furthermore, PWS ARC organoids exhibited a lower frequency of neuronal firing and defective leptin signalling, the hormone responsible for governing feelings of satiety and appetite regulation.^135^ Transcriptome profiling of PWS organoids showed abnormal gene expression patterns. Interestingly, differentially expressed genes found in PWS ARC organoids correlated with those identified in post-mortem hypothalamus samples from PWS patients.^135^ This observation suggested that some transcriptional defects characterizing PWS may persist from the embryonic stage to postnatal development. The transcriptional analysis also highlighted an upregulation of genes linked to immune response and inflammatory processes.^135^ The authors therefore proposed that PWS ARC organoids might favour the infiltration of immune cells and microglia in the hypothalamus. To assess this hypothesis, they transplanted both control and patient ARC organoids into the brains of adult mice. Histological examination of the xenografts conducted 2 months after transplantation revealed a higher presence of infiltrated microglia exhibiting an activated morphology in PWS organoids. This observation aligns with the heightened inflammatory transcriptomic signature observed in PWS organoids.^135^

## Tuberous sclerosis complex

Tuberous sclerosis complex (TSC) is a multisystemic disorder caused by mutation in the *TSC1* (hamartin) or *TSC2* (tuberin) genes.^136^ Proteins produced from the *TSC1* and *TSC2* genes form a heterodimeric complex whose primary function is to inhibit the mTOR kinase.^137^ mTOR signalling plays a crucial role in regulating various essential cellular processes, including protein synthesis, autophagy and energy sensing. Consequently, disruption of the TSC1/TSC2 inhibitory complex leads to increased mTOR activity, which in turn causes alterations in cell growth, differentiation, proliferation and metabolism.^138^ TSC individuals are subject to developing non-malignant hamartomas that can affect their lungs, skin, heart, kidneys and brain.^139^ However, the neurological symptoms presented by TSC patients, which include epileptic seizures, intellectual disability and ASD, remain the most debilitating aspects of the disease.^140^ Most TSC patients develop cortical tubers, which are macroscopic malformed regions within the cerebral cortex characterized by giant cells as well as dysmorphic neurons and astrocytes.^141^ Cortical tubers often become epileptic foci, and an increase in their number is associated with more severe neurological manifestations, such as increased cognitive impairments and behavioural issues.^142^ Additionally, approximately 80% of TSC patients develop benign tumours called subependymal nodules (SEN) along the proliferative areas of brain ventricles, which can potentially progress into subependymal giant cell astrocytoma (SEGA).^139^

Studies using TSC animal models showed various deficits, including changes in neuronal differentiation and morphology.^143,144^ These findings align with observations made in post-mortem tissue from TSC patients.^145^ However, mice with mutations in the *Tsc1* or *Tsc2* genes do not develop cortical tubers, a hallmark of the disease.^146,147^ This inability of rodent models to fully mimic the complete range of neurodevelopmental abnormalities observed in TSC patients confirms that there are certain inherent distinctions in brain development between mice and humans. Blair *et al*.^148^ used cortical organoids derived from TSC2 KO embryonic stem cells to demonstrate that TSC2 loss-of-function led to a bias in neural progenitor cell differentiation towards the glial lineage. This resulted in reduced neuron production and an increased generation of astrocytes within TSC2 KO organoids. Additionally, they observed the presence of dysmorphic neurons and astrocytes in mutant organoids, resembling cells found in cortical tubers in individuals with TSC. Characterization of mTOR signalling in wild-type organoids revealed its suppression during cortical differentiation. However, this pattern of mTOR inhibition was absent in KO organoids, resulting in increased activation of mTOR downstream effectors such as the STAT3 transcription factor known to promote astrocyte differentiation. The findings implied that the inhibition of mTOR might rescue the neurodevelopmental abnormalities induced by TSC2 KO. As such, chronic rapamycin treatment (starting at Day 12 and ending at Day 110 of organoid development) was able to rescue the imbalance between neuron and astrocyte generation, normalize mTOR signalling and correct the aberrant cell morphology.^148^ However, when rapamycin treatment was administrated later (from Day 80 to Day 110) or not throughout the entire organoid differentiation process (from Day 12 to Day 80), it failed to produce the same beneficial effects. This suggested that, although highly unrealistic, sustained prenatal mTOR inhibition may be an effective approach for alleviating some of the neurological symptoms experienced by TSC patients.^148^

The current understanding of TSC pathogenesis, largely derived from research in animal models, proposes that disease onset depends on the inactivation of a second allele of either TSC1 or TSC2 (two-hit model).^139^ However, genetic analyses of post-mortem tissue from TSC patients revealed that the loss of heterozygosity occurs in most SEN and SEGA, but only in a limited number of cortical tubers, challenging the previously mentioned two-hit model.^149^ Moreover, transcriptional analyses have indicated that SEN and cortical tubers share a common cellular origin, but the identity of this specific cell type remains to be determined.^149^ To address these issues, a recent study introduced a method for inducing the development of SEN or cortical tubers in cerebral organoids derived from iPSCs carrying TSC2 mutations.^150^ Indeed, culturing these organoids in a high-nutrient medium promoted the development of SEN, while culturing them in a low-nutrient medium supplemented with neurotrophic factors facilitated the formation of tubers within organoids. Using this method, the authors demonstrated that approximately 98% of cells within the tubers expressed a functional TSC2 allele, challenging the notion that biallelic inactivation is a prerequisite for tuber formation.^150^ Conversely, the characterization of tumour cells within TSC organoids revealed that the second TSC2 allele was predominantly inactivated during tumour progression. This phenomenon does not result from a somatic mutation but rather from a copy-neutral loss of heterozygosity.^150^

Single cell RNA-seq of both tuber-like and tumour-like organoids also revealed an overrepresentation of caudal ganglionic eminence cells, a type of interneuron progenitor.^150^ Subsequent RNA velocity analysis found that the differentiation trajectory of mature interneurons was separated into tumours and tuber-enriched interneurons. These results indicated that the interneuron progenitors overrepresented in TSC organoids follow defined developmental trajectories and diverge into lesion-specific interneuron subtypes. Moreover, a comparison of TSC organoids transcriptomic profiles with scRNAseq data from foetal brains revealed that these cell populations closely resemble caudal ganglionic eminence progenitors emerging around the late gestation period.^150^ Together, these results exposed the limitation of the previously proposed two-hit model of TSC physiology and identified the cell type at the origin of both cortical tubers and SEN.

## Conclusions and perspectives

**Figure 3:**
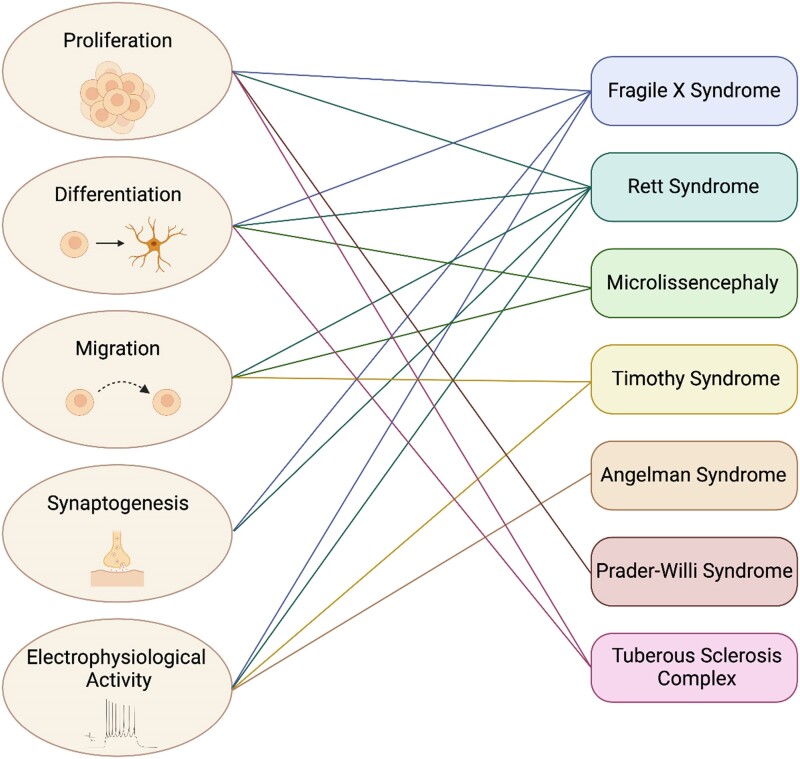
**Connectome-based modelling of neurodevelopmental disorders**. Connectome depicting the manifold cellular defects identified in brain organoids modelling the neurodevelopmental disorders discussed in this review.

Generating brain organoids from human iPSCs offers unprecedented opportunities for studying human brain development. This advancement enables researchers to give insights into stages of human neurodevelopment that were previously inaccessible and has helped to overcome many inherent limitations associated with the use of transgenic animals in the study of NDD. As detailed in this review and listed in Table 1, brain organoids have played a pivotal role in advancing our understanding of NDD. For instance, organoids allowed the identification of pathological mechanisms common to various NDD, such as cellular defects linked to the proliferation, differentiation or migration of specific cell types as well as abnormal synaptogenesis or electrophysiological activity (Fig. 3). Furthermore, the ongoing refinement and sophistication of protocols for generating brain organoids will enhance our ability to understand the pathophysiology of these NDD. It will ultimately allow for greater organoid maturation, better modelling of the interactions between different brain regions or between the brain and other organs, and enable the investigation of the roles of specific cellular populations.

**Table 1:** NDD and their major phenotypes characterized in brain organoids

NDD: *Gene*/Locus	Organoid type	Phenotype/major findings
Fragile X syndrome: *FMR1*	Cerebral organoid	Larger organoids containing more cortical plates^65^ Higher number of GFAP+ cells^65^
	Forebrain organoid	Dysregulated progenitor cell proliferation^70,71^ Altered cytoarchitecture and neuronal differentiation/speciation^71^ Increased synapse formation and neuronal excitability^71^ Identification of human-specific FMRP RNA targets^71^ PI3K inhibitors, but not mGluR5 inhibitors normalized neurodevelopmental defects^71^ Increased expression of sAPPα^67^
Rett syndrome: *MECP2*	Cerebral organoid	Abnormal progenitor population size and proliferation^84,86^ Altered cytoarchitecture and neuronal differentiation^84,86^ Treatment with valproic acid normalizes progenitor proliferation, neuron production and gene expression^84^
	Cortical organoid	Abnormal progenitor population size and proliferation^85,87^ Dysregulated cell cycle progression and distorted mitotic spindle orientation^85^ Altered cytoarchitecture and neuronal differentiation^85,87^ Increased production of astrocytes^85^ Reduced synapse formation and altered electrophysiological activity^84,87^ Inhibition of TGF-β/BMP pathway restores neuronal specification and reduces astrocyte generation^85^ Nefiracetam and PHA 543613 treatments partially normalize gene expression and electrophysiological activity^93^
	Ganglionic eminence + cortical fusion organoid	Abnormal composition of inhibitory neurons population^91^ Neuronal excitability and atypical neural oscillation pattern^91^ Electrophysiological abnormalities are primarily driven by interneuron dysfunctions^91^ Reduced interneurons migration from the ganglionic eminence to the cortex^87^
Microlissencephaly: 17p13.3	Cerebral organoid	Reduced organoid size^107,108^ Altered RGC mitotic spindle orientation and truncated RGC microtubule network^107,108^ Reduced neuronal migration^108^ Disorganized SVZ^107^ Re-expression of LIS1 or YWHAE partially rescues the neurodevelopmental phenotype^107,108^
Microlissencephaly: *KATNB1*	Cerebral organoid	Disrupted organization of the SVZ^114^ Abnormal neurogenesis and reduced neuronal migration^114^
Angelman syndrome: *UBE3A*	Cortical organoid	Neuronal hyperexcitability characterized by spontaneous burst firing and increased network synchronicity^129^ Increased big potassium (BK) channel protein expression^129^ Paxillin treatment converts burst firing and rescues the increased synchronize activity^129^
	Cerebral organoid	UBE3A nuclear expression is dynamically regulated during neural development in control organoids^130^ Abnormal subcellular distribution of UBE3A in various cell type during organoid maturation^130^ Increased expression of UBE3A-AS and reduced expression of UBE3A mRNA in organoids suggest abnormal imprinting^130^
Prader-Willi syndrome: 15q11.2-q13	Arcuate nucleus organoid	Neurodevelopmental abnormalities marked by the presence of oversized organoids and irregular neural cell population composition^135^ Reduced frequency of neuronal firing^135^ Defective leptin signalling^135^ Good correlation between expression defects in organoids and hypothalamus samples from PWS patients^135^ Transplanted organoids in brain of adult mice exhibit higher number of infiltrated microglia with an activated morphology^135^
Timothy syndrome: *CACNA1C*	Transplanted cortical organoid	Immature neuron morphology with increased dendrites branching^119^ Elevated electrophysiological activity^119^
	Ganglionic eminence + cortical fusion organoid	Abnormal interneuron migration pattern^120^ Reduced saltation length in TS interneurons results from calcium-dependent mechanisms^121^ Increased saltation frequency in TS interneurons is normalized by GABAa inhibitors^121^
Tuberous sclerosis syndrome: *TSC1*/*TSC2*	Cortical organoid	NPC differentiation bias towards the glial lineage^148^ Dysmorphic neurons and astrocytes^148^ Abnormal regulation of mTOR signalling during organoid differentiation^148^ Chronic and sustained mTOR inhibition rescues neurodevelopmental deficits^148^
	Tubers and tumours containing cerebral organoid	Tubers and tumours within cerebral organoids both originate from the same pool of late caudal ganglionic eminence progenitors^150^ Biallelic inactivation is not mandatory for tuber formation^150^ Inactivation of the second TCS2 allele in SEN arises from copy-neutral loss of heterozygosity during tumour progression^150^

NDD = neurodevelopmental disorders; NPC = neural progenitor cells; PWS = Prader-Willi syndrome; RGC = radial glial cells; SEN = subependymal nodules; SVZ = subventricular zone; TS = Timothy syndrome.

However, the use of this relatively new technology comes with several constraints that currently restrict its scope, such as the lack of complex architecture. Indeed, *in vitro* culture conditions impose inherent constraints on organoid development, ultimately limiting their size, maturation and the emergence of higher-order electrophysiological activity.^151^ Moreover, brain organoids are, by default, devoid of vasculature, which is important for neurogenesis.^152^ Brain organoids can be transplanted into animal brains to promote vascularization, but this transplantation is difficult to scale up.^4^ To maximize the utility of brain organoids, vascularized brain organoids have recently been developed by generating brain organoids and blood vessel organoids independently and then fusing them together.^153^ These fusion organoids require further improvement, as they currently lack the scalability to replace traditional organoid cultures; however, they have the advantage of containing functional blood–brain barrier-like structures.

Furthermore, most current differentiation approaches generate brain organoids missing specific cell populations.^152^ Indeed, many studies have shown that the brain contains a rich population of resident immune cells, such as microglia, playing important roles in brain development and homeostasis. Microglia dysfunction is also implicated in the pathogenesis of various brain disorders.^154^ Recent studies have described methods to incorporate mature microglia or microglia precursor cells into brain organoids to generate microglia-containing human brain organoids for the study of brain development.^155^ The ability to replicate the cellular diversity present in the brain is critical for deciphering the complex set of phenotypes observed in NDD. The ongoing development of more advanced organoid models in the coming years holds the promise of enabling groundbreaking discoveries. For instance, organoid modelling interactions between neuronal and non-neuronal components will contribute to a more comprehensive understanding of the pathophysiology of NDD and result in improved care for patients. As such, brain organoids can be used as a platform for the development of new drug candidates to treat NDD by providing insights into their efficacy and toxicological profiles.^156^ Moreover, generating brain organoids with blood–brain-barrier-like structures could also advance the screening of drugs that can cross this barrier.

The use of human iPSCs procures several advantages for the field of developmental biology and its associated disorders. The first is the complete abolition of the ethical issues associated with ESCs, which typically require the use and destruction of a human embryo, even though the use of iPSCs also comes with different ethical concerns, e.g., the possibility of cloning human beings or producing human germ cells.^39,157^Additionally, iPSCs can be modified with genome editing techniques to create advanced model systems.^158,159^ Another appealing aspect is that iPSCs can be derived from any patient and therefore provide the unique opportunity to generate brain organoids from any individual, which is of significant relevance in the realm of personalized medicine. Brain organoids produced from patient-derived iPSCs can be used to develop an optimized and tailored pharmacological intervention or to investigate the contribution of polymorphisms to phenotypic manifestations.

However, the use of patient-derived iPSCs does come with some disadvantages. The current methodologies for producing patient-derived iPSCs are labour-intensive, expensive and take several months to complete, which limits their application in large-scale studies.^160^ It is also important to note that the various reprogramming methodologies currently used, i.e. integrative and non-integrative methods, can impact the genomic integrity, and the functionality of the resulting iPSC lines remains poorly understood.^160^ Fibroblasts, peripheral blood mononuclear cells and urine-derived cells are the somatic cell types routinely used to produce iPSCs. Although they initially appeared functionally similar, iPSCs derived from these different cell types can retain distinct epigenetic characteristics related to their somatic origin.^161^ Moreover, some studies have shown that iPSCs derived from fibroblasts present an increased rate of mutations and chromosomal alterations due to repeated exposure to ultraviolet light.^162,163^ Moreover, there is currently no standardized procedure to validate the integrity, quality and functionality of newly produced iPSC lines.^164^ Finally, even though brain organoids show the same developmental trajectories as human brains, they exhibit strong heterogeneity and variability between organoids in the same dish but also between organoids generated from different patient-derived iPSC lines.^165,166^ This inter-organoid heterogeneity is valuable in recapitulating the individual-to-individual differences in the context of NDD, although the organoid-to-organoid variability can raise some questions. All these issues represent fundamental questions for the field that need to be addressed to reduce variability and ultimately enhance the effectiveness of studies conducted using patient-derived iPSC lines.
